# Association between Toll-like receptor 4 Asp299Gly polymorphism and coronary heart disease susceptibility

**DOI:** 10.1590/1414-431X20176306

**Published:** 2017-08-07

**Authors:** B.W. Wu, J. Zhu, H.M. Shi, B. Jin, Z.C. Wen

**Affiliations:** Department of Cardiology, Huashan Hospital, Fudan University, Shanghai, China

**Keywords:** Toll-like receptor 4, Polymorphism, Coronary heart disease, Meta-analysis

## Abstract

Published data on the association between Toll-like receptor 4 (TLR4) Asp299Gly polymorphism and coronary heart disease (CHD) susceptibility are inconclusive. To derive a more precise estimation of the relationship, a meta-analysis was performed. English-language studies were identified by searching PubMed and Embase databases (up to November 2016). All epidemiological studies were regarding Caucasians because no TLR4 Asp/Gly and Gly/Gly genotypes have been detected in Asians. A total of 20 case-control studies involving 14,416 cases and 10,764 controls were included in the meta-analysis. Overall, no significant associations were found between TLR4 Asp299Gly polymorphism and CHD susceptibility in the dominant model (OR=0.89; 95%CI=0.74 to 1.06; P=0.20) pooled in the meta-analysis. In the subgroup analysis by CHD, non-significant associations were found in cases compared to controls. When stratified by control source, no significantly decreased risk was found in the additive model or dominant model. The present meta-analysis suggests that the TLR4 Asp299Gly polymorphism was not associated with decreased CHD risk in Caucasians.

## Introduction

Epidemiological studies have strongly supported a pivotal role for inflammatory, innate immune, and adaptive immune mechanisms in the pathogenesis of atherosclerosis (1,2). Although the importance of Toll-like receptors (TLRs) in antimicrobial responses is established, the role in atherosclerotic disease is not well understood (3,4). TLR4 is predominantly known for its role as an important mediator of innate immune response and has been implicated in the initiation, progression, and plaque destabilization stages of atherosclerosis (5). It is logical to consider that TLR4-mediated signaling might be a potential target for intervention in the initiation and progression of coronary heart disease (CHD).

Variants in the gene encoding TLR4 may affect the development of atherosclerosis accompanied by an impaired signal transduction, but the responsible polymorphisms remain inconclusive (6,7). A single nucleotide polymorphism TLR4 Asp299Gly (rs4986790) has been reported to be associated with lower levels of proinflammatory serum markers, many of which have been implicated in atherosclerosis. Published studies on TLR4 Asp299Gly polymorphism and CHD susceptibility have generally revealed conflicting data, partially because of the possible weak effect of the polymorphism and the relatively small sample size in individual studies. Therefore, we performed a meta-analysis of the eligible studies to derive a more precise estimation of the association.

## Material and Methods

### Search strategy

This study was carried out and reported in agreement with the PRISMA guidelines for systematic reviews and meta-analyses (8). Each study was approved by the respective Institutional Ethics Committee. All patients had given written informed consent prior to study inclusion. Studies were identified by search of Medline and Embase databases using both electronic and manual search strategies. The comprehensive literature search was performed in October 2016 and updated in November 2016 with a verification search for any new studies. We combined search terms for Toll-like receptor 4, polymorphism and coronary heart disease, and we restricted our search to studies that were published in English. When the studies were duplicated or overlapped, we included the most recently published studies in the final analysis.

### Inclusion criteria

We independently evaluated eligible articles on the basis of the following inclusion criteria: 1) evaluation of TLR4 Asp299Gly polymorphism and CHD susceptibility, 2) case-control studies, and 3) sufficient published data for estimating an odds ratio (OR) with 95% confidence interval (CI).

### Data extraction

Two authors (B.W. Wu and J. Zhu) independently extracted data from all eligible studies fulfilling the inclusion criteria. Disagreement was resolved by discussion between the two authors. If these two authors could not reach a consensus, another author (H.M. Shi) was consulted and a final decision was made by the majority of the votes. Data extraction included the first author’s surname, publication year, origin region, matching criteria, demographic data, and genotyping method. For data not provided in the main text, the required information was obtained in part from supplementary online appendixes.

### Statistical methods

Cochrane collaboration meta-analysis review methodology was used for this study (9). ORs with 95%CI were used to assess the strength of association between TLR4 Asp299Gly polymorphism and CHD risk. The pooled ORs were performed for additive model and dominant model. The heterogeneity across trials was evaluated. P-values less than 0.10 for the Q test indicated statistical heterogeneity among studies, and the overall effect estimate was calculated by the random-effect model. Otherwise, the fixed-effect model was used. All statistical tests were performed with RevMan version 4.2.2 available free from Cochrane Collaboration (http://www.cochrane.org/cochrane/hbook/htm).

## Results

### Study identification

A total of 324 unique citations were identified by our search strategy. After the initial screening, 56 potentially relevant articles were selected for further review. Among these, 36 articles were excluded according to the inclusion criteria. Overall, 20 case-control studies involving 14,416 cases and 10,764 controls were included in the meta-analysis (10–29).

### Study characteristics

Table 1 presents the characteristics of the 20 case-control studies published between 2003 and 2015. All molecular epidemiological studies were regarding Caucasians in the present meta-analysis because no TLR4 Asp/Gly and Gly/Gly genotypes were detected in Asians (30,31). Controls were mainly matched for sex and age, nine studies were population-based, and eleven studies were hospital-based.

**Table 1.** Main characteristics of all case-control studies included in the meta-analysis.

Authors	Year	Region	Matching criteria	Cases	Controls	Case/Control	Genotyping method
Ameziane et al. (10)	2003	France	Age and sex	MI/UA	Healthy subjects	183/216	TaqMan
Balistreri et al. (11)	2004	Italy	Age	MI	Healthy subjects	105/127	AS-PCR
Berg et al. (12)	2009	Norway	–	CHD	CHD-free subjects	130/100	PCR-RFLP
Boekholdt et al. (13)	2003	Netherlands	Age	MI	Non-MI subjects	312/343	AS-PCR
Džumhur et al. (14)	2012	Croatia	Age and sex	MI	Healthy subjects	119/120	TaqMan
Edfeldt et al. (15)	2004	Sweden	Age and sex	MI	Healthy subjects	1172/1517	TaqMan
Enquobahrie et al. (16)	2008	USA	Age and sex	MI	Non-MI subjects	848/2682	AS-PCR
Golovkin et al. (17)	2014	Russia	Age and sex	CHD	Healthy subjects	702/300	TaqMan
Guven et al. (18)	2015	Turkey	–	CHD	CHD-free subjects	150/150	PCR-RFLP
Hamann et al. (19)	2005	UK	Age and sex	CHD	Healthy subjects	388/189	TaqMan
Hernesniemi et al. (20)	2006	Finland	–	CHD	CHD-free subjects	333/299	AS-PCR
Holloway et al. (21)	2005	UK	Age	MI	Non-MI subjects	586/492	ARMS-PCR
Koch et al. (22)	2006	Germany	–	MI	CHD-free subjects	3657/1211	TaqMan
Kolek et al. (23)	2004	USA	–	CHD	CHD-free subjects	1375/519	TaqMan
Martinez-Rios et al. (24)	2013	Mexico	–	ACS	Healthy subjects	457/283	TaqMan
Morange et al. (25)	2004	France/Ireland	Age	CHD	CHD-free subjects	247/490	AS-PCR
Nebel et al. (26)	2007	Germany	Age	MI	Healthy subjects	606/323	TaqMan
O’Halloran et al. (27)	2006	Ireland	–	CHD	Healthy subjects	1598/386	AS-PCR
Yang et al. (28)	2003	UK	–	CHD	CHD-free subjects	1078/322	ARMS-PCR
Zee et al. (29)	2005	USA	Age and smoking	MI	CHD-free subjects	370/695	AS-PCR

ARMS: amplification refractory mutation system; AS-PCR: allele-specific polymerase chain reaction; CHD: coronary heart disease; ACS: acute coronary syndrome; MI: myocardial infarction; RFLP: restriction fragment length polymorphism; UA: unstable angina; UK: United Kingdom, USA: United States of America.

### Main results

**Figure 1.**
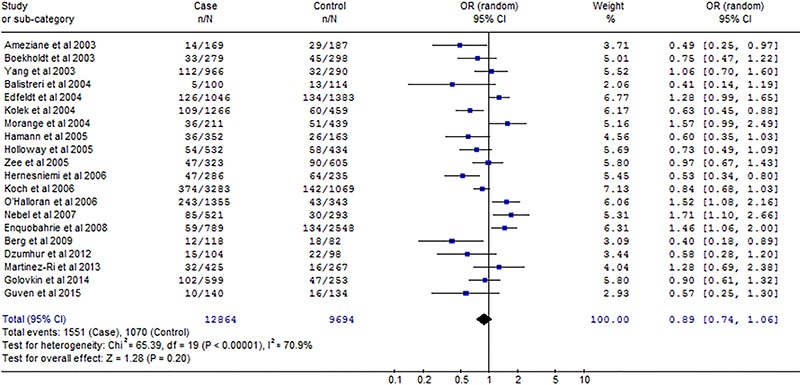
Cumulative odds ratio of TLR4 Asp299Gly polymorphism in patients with coronary heart disease compared with controls in dominant model (OR=0.89; 95%CI=0.74 to 1.06; P=0.20). See Table 1 for reference numbers of cited studies.

Table 2 presents the main results of pooled ORs in the meta-analysis. Overall, no significant associations were found between TLR4 Asp299Gly polymorphism and CHD susceptibility in the dominant model (OR=0.89; 95%CI=0.74 to 1.06; P=0.20, Figure 1) after the meta-analysis. The heterogeneity test indicated statistically significant results (I^2^=70.9%; P<0.00001). In the subgroup analysis by CHD, non-significant associations were found. When stratified by control source, no significantly decreased risk was found in dominant model.

**Table 2.** Summary of pooled ORs according to TLR4 Asp299Gly polymorphism in coronary heart disease patients.

Comparison	Study	Asp/Gly *vs* Asp/Asp		Gly/Gly *vs* Asp/Asp		Dominant model	
		OR (95%CI)	P value	OR (95%CI)	P value	OR (95%CI)	P value
Total	20	0.89 (0.71; 1.13)	0.35	1.12 (0.60; 2.07)	0.72	0.89 (0.74; 1.06)	0.20
Category							
ACS (MI/UA)	11	0.90 (0.68; 1.20)	0.48	0.94 (0.42; 2.08)	0.88	0.95 (0.77; 1.19)	0.68
CHD (mixed)	9	0.86 (0.56; 1.32)	0.49	1.44 (0.54; 3.85)	0.47	0.82 (0.60; 1.11)	0.20
Control source							
Hospital	11	0.78 (0.46; 1.30)	0.34	1.28 (0.52; 3.17)	0.59	0.82 (0.65; 1.05)	0.11
Population	9	0.93 (0.70; 1.24)	0.64	0.99 (0.42; 2.30)	0.98	0.97 (0.75; 1.26)	0.83

ACS: acute coronary syndrome; CHD: coronary heart disease; MI: myocardial infarction; UA: unstable angina; OR: odds ratio; CI: confidence interval.

**Figure 2.**
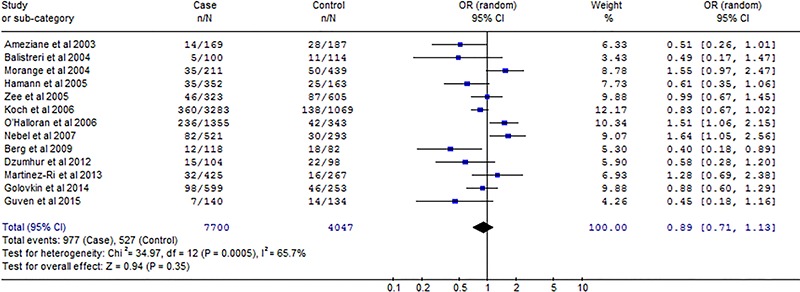
Forest plot for the meta-analysis of the association between TLR4 Asp299Gly polymorphism (Asp/Gly *vs* Asp/Asp) and coronary heart disease from 13 case-control studies (OR=0.89; 95%CI=0.71 to 1.13; P=0.35). See Table 1 for reference numbers of cited studies.

**Figure 3.**
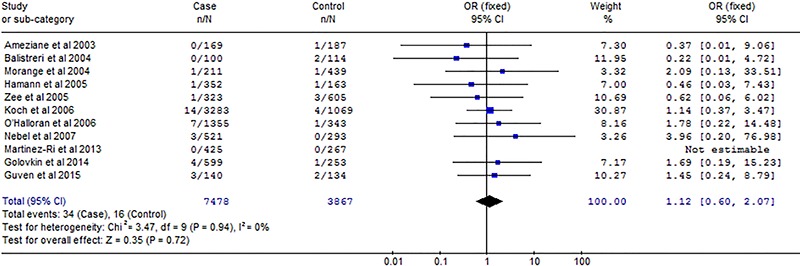
Summary plot of TLR4 Asp299Gly polymorphism (Gly/Gly *vs* Asp/Asp) in patients with coronary heart disease compared with controls (OR=1.12; 95%CI=0.60 to 2.07; P=0.72). See Table 1 for reference numbers of cited studies.

As depicted in Figure 2, no significantly decreased risk was found in the meta-analysis of TLR4 Asp299Gly polymorphism (Asp/Gly *vs* Asp/Asp) from 13 case-control studies (OR=0.89; 95%CI=0.71 to 1.13; P=0.35). As shown in Figure 3, no statistically significant associations were found in the relationship of TLR4 Asp299Gly polymorphism (Gly/Gly *vs* Asp/Asp) in patients with CHD compared with controls (OR=1.12; 95%CI=0.60 to 2.07; P=0.72). In the subgroup analysis by CHD and control source, non-significant associations were found in additive model.

### Sensitivity analysis

A single study involved in the meta-analysis was deleted each time to reflect the influence of the individual data-set to the pooled ORs, and the corresponding ORs were not significantly altered, indicating that our results were statistically robust (32).

### Publication bias

Begg’s funnel plot and Egger’s test were performed to access the publication bias of the studies. The funnel-plot analysis indicated that no significant publication bias was detected to influence the results of this meta-analysis.

## Discussion

Atherosclerosis, although closely related to lifestyle and environmental factors, is also influenced by the complex patterns of inheritance (33,34). In the past decade, researchers have focused on numerous polymorphisms and mutations in genes that are related to atherosclerosis. However, the contribution of TLR4 to the development of CHD has been less well characterized. The major limitation of current genetic studies, applied to multifactorial diseases, is the lack of phenotypic markers identifying patient subgroups who may have a different prevalence of specific genetic or environmental factors. For this reason, our meta-analysis was performed to investigate the association between TLR4 Asp299Gly polymorphism and CHD.

The present study found non-significant associations between TLR4 Asp299Gly polymorphism and CHD susceptibility in 20 case-control studies. Considering the complex nature of atherosclerosis, it suggests that TLR4 Asp299Gly polymorphism has only a minor impact on the pathogenesis of CHD.

Compared with a previous meta-analysis (35), we included more case-control studies and performed sub-group analyses by stratification according to types of CHD and source of controls. In view of the negative results, some limitations of this meta-analysis should be acknowledged. First, the meta-analysis was not performed on individual patient data, so pre-specified data were partly extracted from studies for analysis. Second, as no TLR4 Asp/Gly and Gly/Gly genotypes have been detected in Asian populations, there was no study representing the Asian population. Third, the potential heterogeneity among trials, due to varying inclusion criteria, definition of variables, and different genotyping methods, also cannot be disregarded.

In conclusion, the present meta-analysis suggested that TLR4 Asp299Gly genetic polymorphism is not involved in the pathogenesis of CHD in Caucasians. However, despite the negative results, a causal relationship may exist in the development of atherosclerosis. Well-designed studies with larger samples should be conducted to confirm the results. Moreover, further studies estimating the effect of gene-gene and gene-environment interactions may eventually provide a better and more comprehensive understanding of the association between TLR4 Asp299Gly polymorphism and CHD susceptibility.
